# Applying Resistant Starch to Improve the Gel and Water Retention of Reduced-Fat Pork Batter

**DOI:** 10.3390/gels10050347

**Published:** 2024-05-19

**Authors:** Chun Xie, Guang-Hui Liu, Ming-Hui Liang, Si-Han Li, Zhuang-Li Kang

**Affiliations:** 1 School of Pharmacy, Shangqiu Medical College, Shangqiu 476100, China; lgh1178@163.com (G.-H.L.); liangminghui123@126.com (M.-H.L.); sqyzjwczyrd2022@163.com (S.-H.L.); 2 Engineering Research Center for Huaiyang Cuisine of Jiangsu Province, College of Tourism and Culinary, Yangzhou University, Yangzhou 225127, China; 008107@yzu.edu.cn

**Keywords:** resistant starch, pork batter, emulsion stability, texture, rheology property

## Abstract

Emulsified meat products contain high animal fat content, and excessive intake of animal fat is not good for health, so people are paying more and more attention to reduced-fat meat products. This study investigated the impact of varying proportions of pork back-fat and/or resistant starch on the proximate composition, water and fat retention, texture properties, color, and rheology characteristic of pork batter. The results found that replacing pork back-fat with resistant starch and ice water significantly decreased the total lipid and energy contents of cooked pork batter (*p* < 0.05) while improving emulsion stability, cooking yield, texture, and rheology properties. Additionally, when the pork back-fat replacement ratio was no more than 50%, there was a significant increase in emulsion stability, cooking yield, hardiness, springiness, cohesiveness, chewiness, and L* and G’ values (*p* < 0.05). Furthermore, resistant starch and ice water enhanced myosin head and tail thermal stability and increased G’ value at 80 °C. However, the initial relaxation times significantly decreased (*p* < 0.05) and the peak ratio of P_21_ significantly increased from 84.62% to 94.03%, suggesting reduced fluidity of water. In conclusion, it is feasible to use resistant starch and ice water as a substitute for pork back-fat in order to produce reduced-fat pork batter with favorable gel and rheology properties.

## 1. Introduction

Emulsified meat products are favored by many people because of their high nutritional value, delicious flavor, and convenient consumption [1]. Fat, as a crucial nutrient in meat products, not only provides essential metabolic energy and increases satiety, but also enhances the stability of the emulsion product, resulting in tender and juicy meat with excellent flavor [2]. Traditional meat products typically contain 15–30% animal fat rich in cholesterol and saturated fatty acids. Excessive consumption of animal fat can lead to hypertension, obesity, and cardiovascular diseases, posing adverse effects on human health [3,4]. Thus, reduced-fat meat products have garnered significant attention from people [5]. Currently, numerous studies have explored the use of starch or a combination of starch and vegetable oil partially or completely instead of animal fats in meat products [6,7]. Zhao et al. [8] discovered that replacing fat with oil-starch improved the gel performance and whiteness of pork meat gels while reducing water mobility as well as total fat and saturated fatty acid content. Dobson et al. [9] reported synergistic interactions between rapidly swelling waxy starch and pea protein isolate, which could serve as the foundation for plant-based meat analogues. However, there is limited research on using resistant starch to substitute animal fat.

Resistant starch is a general term for starch and its degradants that are not absorbed by the small intestine of healthy individuals. It is an insoluble dietary fiber and exists in a variety of starchy foods. Resistant starch can be fermented in the large intestine by gut microbes to produce a variety of short-chain fatty acids (such as lactic acid and succinic acid) and a variety of gases [10,11]. Resistant starch and its metabolites can play a variety of physiological functions in the human body, such as improving gastrointestinal function, preventing a variety of intestinal diseases, preventing and controlling diabetes, reducing glycemic index and cholesterol, anti-tumor, regulating immunity, increasing beneficial bacteria, and promoting mineral absorption, so as to promote human health [12,13]. Therefore, resistant starch, as a new type of dietary fiber, has become the focus of food research, such as reduced-fat and low-carbohydrate foods. Wang et al. [14] found that resistant corn starch increases the water retention and gel strength of myosin gel by increasing storage modulus, inducing the β-sheet transited to α-helix. Sarteshnizi et al. [15] showed that using resistant starch and β-glucan can produce the prebiotic sausage. However, there are few studies on the use of resistant starch to replace animal fat. Therefore, the aim of this research work was to study the influences of proximate composition, emulsion stability, texture properties, water distribution, and rheology characteristics of pork batter when using resistant starch and ice water instead of pork back-fat and find a new way to produce reduced-fat pork batter.

## 2. Results and Discussion

### 2.1. Proximate Composition and Energy Analysis

The changes in proximate composition and energy analysis of cooked pork batters with varying proportions of pork back-fat and/or resistant starch are presented in Table 1. The moisture of the cooked pork batter significantly increased (*p* < 0.05) with the increase in the starch and ice water contents, except the samples of T3 and T4, and the total lipid and energy contents significantly decreased (*p* < 0.05). The high water content of cooked pork batter indicated that the retention of added water of pork batter increased after the addition of resistant starch. This suggests that resistant starch may have good water retention and stickiness, which can absorb and retain water in the pork batter during cooking, thereby increasing the moisture content and decreasing the total lipid and energy contents [16,17]. At the same time, because resistant starch does not contain protein, the protein of pork batter revealed a decreasing trend with the increase in the starch, but the samples of T1, T2, and T3 were not significantly different (*p* > 0.05); and there were no significant differences in ash content, either (*p* > 0.05).

### 2.2. Emulsion Stability

Emulsion stability is an important index to evaluate the water and oil retention of pork batter. The changes in emulsion stability of cooked pork batters with varying proportions of pork back-fat and/or resistant starch are presented in Table 2. The TR and WR significantly decreased (*p* < 0.05) with the increase in the starch and ice water contents, except for the sample of T4. It is well known that starch is a combination of two different macromolecules inside granules with a specific crystallinity (macromolecular organization) before gelatinization, the resistant starch should even be more complex, since the macromolecular organization does not allow the entrance of enzymes for hydrolysis, and it contains a lot of hydroxyls, and a hydroxyl is a hydrophilic group, so it has water absorption, leading to improved emulsion stability of pork batter [18]. While the FR of T1 and T2 showed no significant differences (*p* > 0.05), they then significantly decreased (*p* < 0.05) with the increase in the starch and ice water contents; it is a reason that the addition of pork back-fat decreased. Additionally, resistant starch has a certain ability to emulsify, increasing the number of resistant starch acting as the emulsifier in the pork batter, and then more oil droplets are wrapped by resistant starch [8]. In addition, the reduction in pork back-fat is also conducive to more salt-soluble protein used to construct the meat substrate, improving the water and oil retention performance of pork batter. Thus, in the pork batter, as the starch can absorb and hold water during the processing, the amount of water that muscle protein needs to maintain decreases. The other batter, using resistant starch and water to replace the pork back-fat, led to the lipid content being decreased, the result was in favor of improving the emulsion stability of pork batter.

### 2.3. Cooking Yield

**Figure 1:**
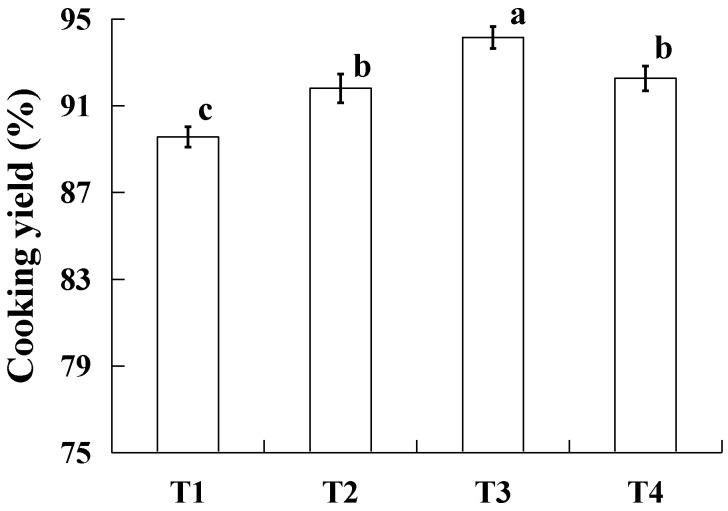
The cooking yield (%) of raw pork batters with varying proportions of pork back-fat and/or resistant starch. T1 contained 40 g ice water and 40 g pork back-fat; T2 contained 3 g resistant starch, 47 g ice water, and 30 g pork back-fat; T3 contained 6 g resistant starch, 54 g ice water, and 20 g pork back-fat; T4 contained 9 g resistant starch, 61 g ice water, and 20 g pork back-fat. Each value represents the mean ± SE, *n* = 4. ^a–c^ Different parameter superscripts indicate significant differences (*p* < 0.05).

During heating, pork batter undergoes protein denaturation and fat melting, resulting in a loss of water and fat. The extent of this loss is closely associated with economic profitability. As shown in Figure 1, adding resistant starch and ice water contents significantly increased (*p* < 0.05) the cooking yield of the pork batter; the sample of T3 exhibited the highest cooking yield, while there were no significant differences (*p* > 0.05) between the samples of T2 and T4. This finding is consistent with the results of emulsion stability (Table 2). It is possible that when the fat replacement ratio was 50%, the fat can be easier to disperse, enhancing the stability. Thus, pork batter showed the best combination ability of water and fat, and the cooking loss was the minimum. Meanwhile, due to the decrease in salt-soluble protein in emulsified pork back-fat, it is beneficial to form cooked batter with good gel structure and improve its water and oil retention performance. In the other batter, the incorporation of resistant starch can enhance electrostatic forces and hydrogen bonds between protein and starch, thereby improving water and oil retention in the blended gel [14,19]. When replacing fat at a ratio exceeding 50%, however, the cooking yield of pork batter begins to decrease due to limitations in water and fat retention capacity within the mixture [20]. Therefore, utilizing a mixture prepared from starch and ice water to replace part of the pork back-fat can effectively increase cooking yield.

### 2.4. Color

The color parameters of cooked pork batter were influenced by the level of fat and the formulation of the batter. The colors of pork batters with varying proportions of pork back-fat and/or resistant starch are shown in Table 3. Compared with the sample of T1, the L* and a* values of pork batters with resistant starch significantly increased (*p* < 0.05), while the b* value significantly decreased (*p* < 0.05). Additionally, the L* value of pork batter significantly increased (*p* < 0.05) when the fat replacement ratio was no more than 50%, resulting in the sample of T3 having the highest L^*^ value, with no significant differences in L* values between T2 and T4 (*p* > 0.05). Furthermore, there were no significant differences in a* and b* values (*p* < 0.05). This can be attributed to enhanced water and fat retention capacity in pork batter due to additional resistant starch (Table 2 and Figure 1), leading to an increase in moisture on the surface favorable for light refraction, thus increasing the L* value of pork batter [21]. Conversely, excessive replacement ratios of resistant starch and ice water led to an increase in water content which disrupted gel network formation in cooked pork batter, leading to a decrease in L* value [22].

### 2.5. Texture Properties

The texture of meat products is a key factor in determining consumer acceptance, and can be influenced by various factors. Table 4 presents the texture properties of pork batters containing different proportions of pork back-fat and/or resistant starch. Compared with the sample of T1, adding resistant starch led to a significant increase (*p* < 0.05) in hardness, springiness, cohesiveness, and chewiness in the pork batter. This can be attributed to the gelation process of resistant starch at high temperatures, which causes an expansion of starch particles and results in changes to the structural properties of the gel-like substances formed [23,24]. Since the resistant starch absorbs water from the surrounding environment during heating, the expanded resistant starch puts pressure on the gel matrix, resulting in an increase in hardness, springiness, cohesiveness, and chewiness [25]. Similarly, previous study has shown that adding resistant corn starch enhances gel strength in chicken breast myosin [14]. Furthermore, when pork back-fat was replaced by no more than 50%, there was a significant increase (*p* < 0.05) in hardness, springiness, cohesiveness, and chewiness observed in the samples of T3 compared to other samples. However, there were no significant differences (*p* > 0.05) between the samples of T2 and T4 except for chewiness. In the gelatinization process, the expansion of starch particles reaches 50~100 times the original volume. Furthermore, the starch does not bind to the muscle protein and only acts as a filler in the gel [8]. Excessive addition of resistant starch and ice water can destroy the formed gel structure.

### 2.6. Dynamic Rheological

**Figure 2:**
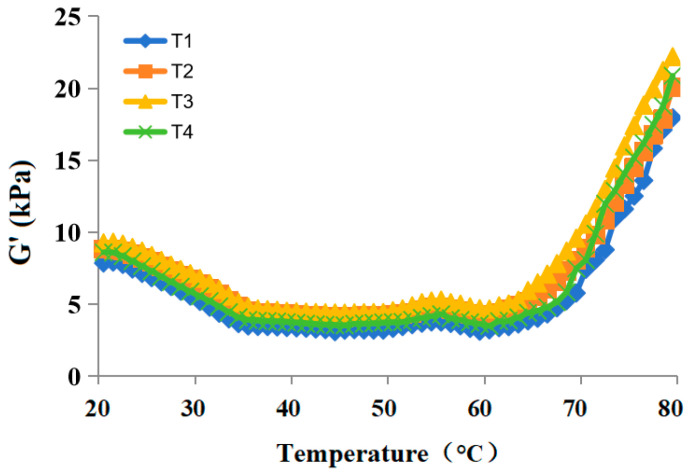
The storage modulus (G’) of raw pork batters with varying proportions of pork back-fat and/or resistant starch. T1 contained 40 g ice water and 40 g pork back-fat; T2 contained 3 g resistant starch, 47 g ice water, and 30 g pork back-fat; T3 contained 6 g resistant starch, 54 g ice water, and 20 g pork back-fat; T4 contained 9 g resistant starch, 61 g ice water, and 20 g pork back-fat.

The changes in G’ of raw pork batters with varying proportions of pork back-fat and/or resistant starch from 20 to 80 °C are depicted in Figure 2. All samples exhibited similar trends, characterized by three distinct stages. Because resistant starch has a better water absorption capacity, the initial G’ values of raw pork batters containing resistant starch were higher than those of sample T1, with T3 displaying the highest value (9.27 kPa). During the first stage, a decrease in G’ values was observed due to the dissolution, swelling, and folding of myofibrillar proteins alongside the melting of pork back-fat as the temperature increased from 20 to 46 °C (T1) or 47 °C (T2, T3, and T4), respectively [26]. Subsequently, in the second stage, a gradual increase in G’ values occurred from 47 to 56 °C (T1) or from 48 to 57 °C (T2, T3 and T4), attributed to enhanced protein–protein interactions resulting from the cross-linking of pork myosin head, leading to weak gel formation at elevated temperatures. This was followed by a slow decrease in G’ values from 57 to 61 °C (T1) or from 58 to 62 °C (T2, T3, and T4), caused by the degeneration of myosin tail and the disruption of the formed weak gel structure [27]. In the third stage, a rapid increase in G’ values was observed as temperature increased from 62 to 80 °C (T1) or from 63 to 80 °C (T2, T3, and T4), indicative of viscoelastic heat-induced gel formation due to aggregation of protein and gel formation resulting in the transformation of sol into elastic colloid [28,29]. Notably, the denaturation temperature of myosin head and tail from the sample of T1 was lower than the pork batters with resistant starch, suggesting that the addition of resistant starch can enhance the thermal stability of the myosin head and tail. Meanwhile, the G’ values of raw pork batters with resistant starch were larger than the sample of T1 during heat processing. This observation may be attributed to that the starch is heated in pork batter until the micellar structure completely collapses, and the starch molecules form a single molecule and become a solution state surrounded by water, and starch molecules are chains or even branches that pull together; the result is a sticky paste solution [30,31], which can effectively fill the gel network and make its structure more compact, thereby improving the gel network structure and elasticity [32,33], leading to the G’ values increased. Furthermore, when the pork back-fat substitution ratio was more than 50%, the G’ values were decreased, which may be because more water was added. Therefore, the batter with a high pork back-fat substitution ratio showed low elasticity.

### 2.7. Low-Filed NMR

Low-field NMR can provide valuable insights into the water holding characteristics of pork batter, which offers a means to analyze water distribution and mobility [34,35]. The T2 relaxation time serves as an indicator of the degree of water immobilization within pork batter, while the peak area proportion reflects water dynamics in different states [36]. The initial relaxation time and the peak ratio of pork batters with varying proportions of pork back-fat and/or resistant starch are shown in Table 5. Three distinct peaks were observed within the range of 0.01 ms to 1000 ms: T_2b_, T_21_, and T_22_. Notably, T_2b_ corresponds to bound water, constituting about 1–4% of the total water content in pork batter. This fraction is tightly associated with polar groups (carboxyl and amino) via hydrogen bonding, binding to proteins, and macromolecular components. The corresponding relaxation time falls within the range of approximately 0–10 ms [37]. On the other hand, T_21_ represents immobile water with a relaxation time ranging from approximately 30–100 ms, meaning that the water was loosely bound to the sol matrix of pork batter [38]. Lastly, T_22_ signifies free water dispersed outside cells or on meat surfaces; its onset relaxation time spans roughly from 100–1000 ms [39]. Compared with the sample of T1, the initial relaxation times of T_2b_, T_21_, and T_22_ from cooked pork batter with resistant starch significantly increased (*p* < 0.05). The T2 can reflect the fluidity of water laterally, and a longer T2 means a higher water fluidity [40]. Thus, the result indicated that the water in the pork batter was tightly bound, resulting in reduced water mobility when added resistant starch [41]. Additionally, the initial relaxation time of T_2b_ from pork batter with resistant starch showed no significant differences (*p* > 0.05) with the increase in resistant starch and ice water, but the initial relaxation times of T_21_ and T_22_ significantly decreased (*p* < 0.05) when the fat replacement ratio was no more than 50%, resulting in the samples of T3 having the shortest initial relaxation times of T_21_ and T_22_, and the samples of T2 and T4 showed no significant differences (*p* > 0.05). This finding is consistent with the results of emulsion stability and cooking yield (Table 2 and Figure 1). This is possibly due to the increase in the water holding capacity of pork batter when the fat replacement ratio was no more than 50%, meaning that the formation of a high-quality cooked pork batter during processing [38]. Meanwhile, there was no significantly difference (*p* > 0.05) in P_2b_ in all samples. Compared with the sample of T1, the peak ratio of P_21_ from cooked pork batter with resistant starch significantly increased (*p* < 0.05), but the P_22_ significantly decreased (*p* < 0.05). This finding is consistent with the results of initial relaxation times (Table 5). Furthermore, the peak ratio of P_21_ from pork batter with resistant starch significantly increased (*p* < 0.05) when the fat replacement ratio was no more than 50%, resulting in the sample of T3 having the largest peak ratio of P_21_, and the samples of T2 and T4 showed no significant differences (*p* > 0.05). On the contrary, the peak ratio of P_22_ from pork batter with resistant starch significantly decreased (*p* < 0.05) when the fat replacement ratio was no more than 50%, resulting in the samples of T3 having the smallest peak ratio of P_22_, and the samples of T2 and T4 showed no significant differences (*p* > 0.05). The results showed that the appropriate amount of resistant starch was beneficial in reducing the fluidity of water, but the excessive amount destroyed the gel structure and enhanced the fluidity of water.

## 3. Conclusions

The result shown that the replacement of pork back-fat with resistant starch and ice water had a significant impact on the technical characteristics, rheological properties, and water distribution of pork batter. The moisture and resistant starch content in the pork batter increased significantly, while the levels of pork back-fat and energy decreased significantly. Additionally, there was an improvement in gel strength and rheological properties with the addition of resistant starch and ice water. Furthermore, replacing 50% of pork back-fat with resistant starch and ice water (T3) resulted in the highest emulsion stability, cooking yield, hardness, springiness, cohesiveness, chewiness, L* value, and G’ value. However, when 75% of pork back-fat was instead of resistant starch and ice water, it negatively affected the gel structure leading to reduced gel strength and rheological properties along with increased fluidity. Overall, substituting 50% of pork back-fat with resistant starch and ice water could produce a higher cooking yield as well as improved texture properties for pork batter. It also provided a new idea for the development of new types of reduced-fat and low-energy emulsion meat products.

## 4. Materials and Methods

### 4.1. Raw Materials

Chilled pork lean leg meat (71.19 ± 0.43% water, 19.96 ± 0.65% protein, 1.96% ± 0.19 ash, and 6.05 ± 0.34% fat, pH 5.69 ± 0.01) and pork back-fat (90.75 ± 0.81% fat, 6.22 ± 0.37% water, 0.34 ± 0.09% ash, and 2.35 ± 0.28% protein) were purchased from a local slaughterhouse (Shangqiu, China). The lean meat was ground with a grinder using a 6 mm hole plate (RY-22S, Zhengyuan Precision Machinery (Jiangsu) Co., Ltd., Suzhou, China), then vacuum packaged and stored at −40 °C. The pork back-fat was ground before used within 2 h. Resistant starch (RSII, HI-MAIZE260, pH 5.60, moisture 10.20%, total dietary fiber 54.70%) was purchased from National Starch Company (Westchester, IL, USA). Sodium chloride and white pepper (food grade) were purchased from a local market (Shangqiu, China).

### 4.2. Preparation of Pork Batters

All of pork batters were prepared with 200 g meat, 3.6 g sodium chloride, and 1.5 g white pepper. Therein: T1 contained 40 g ice water and 40 g pork back-fat; T2 contained 3 g resistant starch, 47 g ice water, and 30 g pork back-fat; T3 contained 6 g resistant starch, 54 g ice water, and 20 g pork back-fat; and T4 contained 9 g resistant starch, 61 g ice water, and 20 g pork back-fat. Thus, the resistant starch and ice water mixture were used to replace 0% (T1), 25% (T2), 50% (T3), and 75% (T4) of the pork back-fat in the pork batters, respectively. The production processing was as follows: firstly, put the meat and salt into a grinder (Stephan UMC-5C, Hamburg, Germany), 1500 r/min, chopped for 60 s; following, added half of ice water, 1500 r/min, chopped for 60 s; and then added the remaining ice water, pork back-fat, and/or resistant starch, 2500 r/min, chopped for 90 s; the temperature was not more than 7 °C during the processing. After chopping, 30 g raw batter was put into 50 mL centrifuge tubes and discharge air (1000× *g*, 3 min), and then heated in a water bath at 80 °C for 20 min. The batter was moved 4 °C refrigerator for storage after cooled to room temperature using running water (25 ± 2 °C).

### 4.3. Proximate Composition and Energy

Following the AOAC method, the proximate composition of cooked pork batter was determined, and its energy content was calculated to be 17 kJ/g protein and 37 kJ/g fat according to the method of Southgate and Durnin [42].

### 4.4. Emulsion Stability

Emulsion stability of raw pork batter was measured by the method of Fernandz-Martín et al. [43]. Briefly, 30 g raw sample was centrifuged at 500× *g* (15 min, 4 °C) in a centrifuge tube to eliminate air bubbles. Then, the sample was heated in an 80 °C water bath for 20 min, and after that, removed and cooled to 20 °C with running water. Following, the centrifuge tubes were left inverted on paper towels for 50 min at room temperature to release any exudate. The total fluid release (TR) was indicated as the percentage of the initial sample weight. The water released (WR) was determined from the dry weight content of TR after heating at 105 °C for 16 h. The fat percentage released (FR) was regarded as the difference between TR and WR.

### 4.5. Cooking Yield

After being heated in a water bath at 80 °C for 20 min, the pork batter was cooled using running water and stored at 4 °C for 12 h. The cooking yield was calculated according to the following formula:

Cooking yield (%)=weight of cooked pork batter/weight of raw pork batter× 100%

### 4.6. Color

The whiteness of cooked pork batter was measured using a CR-400 chromometer (Minolta, Tokyo, Japan) with an aperture of 8 mm, a 10^◦^ observer angle, and D65 illuminant. Then, the whiteboard (L* = 96.86, a* = −1.05, b* = −3.73) was calibrated before measurement. 

### 4.7. Texture Profile Analysis

After rest for 2 h at 20 °C, the cooked batter was shaped into a cylindrical (diameter, 15 mm; height, 15 mm), and the texture profile analysis was performed using a texture analyzer with a P/36R probe (Stable Micro System Ltd., Godalming, UK). The pre-test speed was 5.0 mm/s; the test speed was 2.0 mm/s; the post-test speed was 2.0 mm/s; the strain was 50%. The hardness (N), springiness, cohesiveness, and chewiness (N·mm) of the batter were obtained.

### 4.8. Dynamic Rheology

The rheology property of raw batter was measured using a Haake Mars 60 rotary rheometer fitted with a P35TiL plate probe (Thermo Scientific, Waltham, MA, USA). The gap distance between the two plates was set at 1 mm. The storage modulus (G’) from 20 to 80 °C was measured using a rate of 2 °C/min and the frequency of continuous shearing was 0.1 Hz.

### 4.9. Low-Field NMR

According to the report of Kang et al. [32], 2 g cooked pork batter was put in a 25 mm NMR tube, and Low-field NMR was measured using an NMR analyzer (PQ001, Niumag Corporation, Shanghai, China).

### 4.10. Statistical Analysis

The data were analyzed by General Linear Model procedure (SPSS.v.26.0, Chicago, IL, USA), and the significant differences between means (*p* < 0.05) were analyzed using Duncan’s Multiple Range Test.

## Figures and Tables

**Table 1:** Proximate composition and energy analysis of cooked pork batters with varying proportions of pork back-fat and/or resistant starch.

Sample	Moisture (%)	Protein (%)	Total Lipid (%)	Ash (%)	Starch (%)	Energy (kJ/100 g)
T1	65.21 ± 0.53 ^c^	16.03 ± 0.36 ^a^	15.48 ± 1.25 ^a^	3.27 ± 0.23 ^a^	0 ± 0.00 ^d^	845.39 ± 17.43 ^a^
T2	68.3 ± 0.66 ^b^	15.47 ± 0.42 ^a^	11.47 ± 1.36 ^b^	3.29 ± 0.14 ^a^	1.61 ± 0.30 ^c^	710.86 ± 22.66 ^b^
T3	70.24 ± 0.62 ^a^	15.22 ± 0.29 ^ab^	8.19 ± 1.01 ^c^	3.28 ± 0.17 ^a^	3.06 ± 0.26 ^b^	610.98 ± 24.45 ^c^
T4	71.23 ± 0.55 ^a^	14.91 ± 0.44 ^b^	5.69 ± 1.20 ^d^	3.22 ± 0.25 ^a^	5.16 ± 0.32 ^a^	540.4 ± 20.71 ^d^

T1 contained 40 g ice water and 40 g pork back-fat; T2 contained 3 g resistant starch, 47 g ice water, and 30 g pork back-fat; T3 contained 6 g resistant starch, 54 g ice water, and 20 g pork back-fat; T4 contained 9 g resistant starch, 61 g ice water, and 20 g pork back-fat. Each value represents the mean ± SE, *n* = 4. ^a–d^ Different parameter superscripts indicate significant differences (*p* < 0.05).

**Table 2:** The emulsion stability (TR, WR, and FR, %) of raw pork batters with varying proportions of pork back-fat and/or resistant starch.

Sample	TR (%)	WR (%)	FR (%)
T1	10.75 ± 0.62 ^a^	7.13 ± 0.35 ^a^	3.88 ± 0.21 ^a^
T2	8.28 ± 0.49 ^b^	5.06 ± 0.44 ^b^	3.46 ± 0.19 ^a^
T3	6.12 ± 0.55 ^c^	3.62 ± 0.28 ^c^	2.25 ± 0.17 ^b^
T4	7.81 ± 0.58 ^b^	6.48 ± 0.41 ^b^	1.37 ± 0.24 ^c^

TR, Total fluid released; WR, Water released component; FR, fat released component. T1 contained 40 g ice water and 40 g pork back-fat; T2 contained 3 g resistant starch, 47 g ice water, and 30 g pork back-fat; T3 contained 6 g resistant starch, 54 g ice water, and 20 g pork back-fat; T4 contained 9 g resistant starch, 61 g ice water, and 20 g pork back-fat. Each value represents the mean ± SE, *n* = 4. ^a–c^ Different parameter superscripts indicate significant differences (*p* < 0.05).

**Table 3:** The color of cooked pork batters with varying proportions of pork back-fat and/or resistant starch.

Sample	L* Value	a* Value	b* Value
T1	77.52 ± 0.76 ^c^	1.65 ± 0.25 ^a^	7.35 ± 0.31 ^a^
T2	80.18 ± 0.64 ^b^	1.78 ± 0.19 ^a^	8.47 ± 0.27 ^b^
T3	82.61 ± 0.71 ^a^	1.82 ± 0.21 ^a^	8.63 ± 0.29 ^b^
T4	79.71 ± 0.58 ^b^	1.95 ± 0.28 ^a^	8.80 ± 0.38 ^b^

T1 contained 40 g ice water and 40 g pork back-fat; T2 contained 3 g resistant starch, 47 g ice water, and 30 g pork back-fat; T3 contained 6 g resistant starch, 54 g ice water, and 20 g pork back-fat; T4 contained 9 g resistant starch, 61 g ice water, and 20 g pork back-fat. Each value represents the mean ± SE, *n* = 4. ^a–c^ Different parameter superscripts indicate significant differences (*p* < 0.05).

**Table 4:** The texture properties of cooked pork batters with varying proportions of pork back-fat and/or resistant starch.

Sample	Hardness (N)	Springiness	Cohesiveness	Chewiness (N.mm)
T1	53.39 ± 0.82 ^c^	0.802 ± 0.005 ^c^	0.453 ± 0.009 ^c^	19.44 ± 0.39 ^d^
T2	56.07 ± 0.75 ^b^	0.831 ± 0.007 ^b^	0.491 ± 0.008 ^b^	22.78 ± 0.47 ^c^
T3	58.73 ± 0.66 ^a^	0.866 ± 0.007 ^a^	0.534 ± 0.012 ^a^	27.16 ± 0.41 ^a^
T4	56.63 ± 0.79 ^b^	0.845 ± 0.006 ^b^	0.503 ± 0.010 ^b^	24.14 ± 0.32 ^b^

T1 contained 40 g ice water and 40 g pork back-fat; T2 contained 3 g resistant starch, 47 g ice water, and 30 g pork back-fat; T3 contained 6 g resistant starch, 54 g ice water, and 20 g pork back-fat; T4 contained 9 g resistant starch, 61 g ice water, and 20 g pork back-fat. Each value represents the mean ± SE, *n* = 4. ^a–d^ Different parameter superscripts indicate significant differences (*p* < 0.05).

**Table 5:** The initial relaxation time (ms) and the peak ratio (%) of cooked pork batters with varying proportions of pork back-fat and/or resistant starch.

Sample	Initial Relaxation Time (ms)			Peak Ratio (%)		
	T_2b_	T_21_	T_22_	P_2b_	P_21_	P_22_
T1	2.53 ± 0.19 ^a^	63.57 ± 2.46 ^a^	560.72 ± 19.77 ^a^	1.26 ± 0.21 ^a^	84.62 ± 1.16 ^c^	14.41 ± 0.51 ^a^
T2	1.68 ± 0.16 ^b^	55.62 ± 2.37 ^b^	446.25 ± 23.63 ^b^	1.05 ± 0.16 ^a^	89.75 ± 0.95 ^b^	9.92 ± 0.43 ^b^
T3	1.57 ± 0.22 ^b^	42.90 ± 2.88 ^c^	332.61 ± 25.08 ^c^	1.20 ± 0.09 ^a^	94.03 ± 0.92 ^a^	4.35 ± 0.59 ^c^
T4	1.61 ± 0.19 ^b^	53.81 ± 1.91 ^b^	469.08 ± 22.37 ^b^	1.32 ± 0.15 ^a^	90.26 ± 1.08 ^b^	8.67 ± 0.37 ^b^

T1 contained 40 g ice water and 40 g pork back-fat; T2 contained 3 g resistant starch, 47 g ice water, and 30 g pork back-fat; T3 contained 6 g resistant starch, 54 g ice water, and 20 g pork back-fat; T4 contained 9 g resistant starch, 61 g ice water, and 20 g pork back-fat. Each value represents the mean ± SE, *n* = 4. ^a–c^ Different parameter superscripts indicate significant differences (*p* < 0.05).

## Data Availability

The data presented in this study are openly available in article.
